# Implementing WHO-Quality Rights Project in Tunisia: Results of an Intervention at Razi Hospital

**DOI:** 10.2174/1745017902016010125

**Published:** 2020-07-30

**Authors:** Mauro Giovanni Carta, Rym Ghacem, Myriam Milka, Olfa Moula, Nidhal Staali, Uta Uali, Ghassene Bouakhari, Monica Mannu, Rym Refrafi, Souha Yaakoubi, Maria Francesca Moro, Marie Baudel, Simon Vasseur-Bacle, Natalie Drew, Michelle Funk

**Affiliations:** 1 Department of Medical Sciences and Public Health, University of Cagliari, Cagliari, Italy; 2 Razi Hospital, Tunis, Tunisia; 3 Mental Health Departement ,University Hospital Mongi Slim, Tunis, Tunisia; 4 Mailman School of Public Health, Columbia University, New York, NY, USA; 5 Department of Public Health, WHO, Geneva, Switzerland; 6 WHO Collaborating Centre for Mental Health Lille, Lille, France

**Keywords:** WHO Quality rights project, Human rights, Mental health, Psychosocial intervention, Disabilities, Degrading treatment

## Abstract

**Background:**

The aims were: 1) to measure the attitudes of learners (and future trainers) before and after a course on WHO-Quality Rights (QR); 2) to evaluate a psychiatric ward, by previously trained staff on QR, comparing it with a previous evaluation and discussing an improvement plan.

**Methods:**

1) Training sample: 19 subjects (8 males), 41.4±10.6 years, including jurists/lawyers, health professionals, and experts.

The QR team developed the 26-item tool to assess the knowledge and attitudes of participants.

2) Evaluation of quality of care and respect for human rights in the ward was carried out on 20 staff representatives, 20 family members and 20 users with QRToolkit.

**Results:**

1) Learning in QR has partially changed the knowledge and attitudes of trained people.

2) The evaluation shows significant delays in the implementation of the rights advocated by the United Nations Convention on the Human Rights of Persons with Disabilities (CRPD). In Themes 1, 3, 4 and 5, the evaluation shows no differences compared to 2014, but in Theme 2, the level was lower than four years before.

**Conclusion:**

The scarcity of resources due to the economic crisis that Tunisia is going through, cannot be considered the only cause of the delays highlighted. However, it is likely that in a context of uncertainty for the future, scarcity of resources and a decrease in staff (*i.e*., professionals dedicated to psychosocial intervention) may have demotivated the team towards recovery. The improvement in knowledge and attitudes of many staff members after the training may open future positive scenarios.

## 1 INTRODUCTION

The WHO Quality Rights project (QR) [1] aims to implement the United Nations Convention on the Human Rights of Persons with Disabilities (CRPD) (UN 2006) [2, 3], in the field of psychosocial disability. Its purpose is “to improve access to quality mental health and social services and to promote the rights of people with mental health conditions, and psychosocial, intellectual and cognitive disabilities” [1].

Tunisia signed (2007) and ratified (2008) the CRPD [4], aiming to improve the level of respect for the human rights of people with disabilities (including psychosocial ones).

The project “Tunisia and Sardinia in support of the quality of human rights of people with psychosocial disabilities”, funded by the Sardinian Cooperation, aims to support the application of the QR program in Tunisia. This project conducted a pilot intervention to train a group of professionals, human rights experts and people with experience in psychosocial disability, with the principles of CRPD and the use of instruments of the QR project (“ToolKit”) [5] through an intensive one-week course in Tunisia. The purpose was to create the first group of trainers available for future experience.

The participants of the intensive course completed a questionnaire, before and at the end of the course; the questionnaire evaluated the participant's knowledge and attitudes regarding human rights in mental health and the CRPD. The group of training participants then conducted a standardized assessment of Quality of Care focused on compliance with human rights in a Razi Hospital ward in accordance with QR rules and application of the QR ToolKit. The group then discussed the results with the staff of the ward and hypothesized possible implementation plans.

The aim of the study was to measure the knowledge and attitudes of the training participants (and future trainers) before and after the course, present the results of the evaluation of the psychiatric ward, compare them with a similar evaluation conducted four years earlier and discuss the results and differences between the two assessments over time in the light of the state of the country’s public health system and socio-economic conditions.

## 2 METHODS

### 2.1 Setting

The Razi hospital is the only psychiatric hospital in Tunisia. It guarantees 6 beds x 100,000 inhabitants of the country (WHO 2009). It is estimated that Razi Hospital is supported by about 50% of total spending on mental health, which in turn would represent only 1% of total health expenditure, representing 6.4% of the Gross Domestic Product (GDP) [4]. Tunisia's mental health policies were established in 1992 and have undergone some changes thanks to a specific law of 2004 [4]. The guidelines prefigured two components of care, one in the hospital and the other integrating mental health care into primary health care, to guarantee fair access to mental health services for the majority of citizens in the community. The law provides for the development of human resources, protection of users' human rights, support and promotion, quality improvement and a monitoring system in the field of mental health. However, it involves neither users nor families (in contrast to the CRPD), nor does it refer to the methods of financing. Also, the Tunisian legislator has not addressed the problem of reducing the part of psychiatric inpatient care in favor of developing community care, nor have adequate resources been allocated for this purpose.

According to the 2009 WHO report [4], there are 16 public outpatient mental health facilities in the country, of which 13% are for children and adolescents only. These facilities treat around 1.000 users per 100,000 inhabitants of the community (only considering public services) in a year. All outpatient facilities, with the exception of the outpatient clinic connected to the psychiatric hospital, provide follow-up assistance in the community, while there are no mobile mental health clinic teams. In terms of available interventions, few users (less than 20%) have received one or more psychosocial interventions in the last year, given the high number of patients involved and the limited number of services. The data collected in 2009 does not seem to have currently improved. In recent years, staff involved in rehabilitation therapy in Razi Hospital also progressively decreased, including the mental health facilities; it was not sometimes available one psychotropic drug of each principal therapeutic class (antipsychotic, antidepressant, mood stabilizer, anxiolytic and antiepileptic drugs) nor in a nearby pharmacy. With regards to the accessibility to pharmacotherapy (in general), the Tunisian media often complained of shortcomings in the months before the assessment.

Professional staff complains of a gradual decrease in resources that has led to a reduction in the number of interventions in recent years.

There are no community residential facilities available in the country, but only protected homes for people with mental disabilities without family support, whose capacity (two hundred beds) has long been insufficient.

Organizations of professionals and scientific societies have repeatedly expressed the need for a renewal low related to mental health as well as the recruitment of more human resources in mental health and better availability of medications.

For this reason, it appeared useful to compare the data of this study with the results of an evaluation carried out in 2014 in the same hospital [5].

### 2.2 Design of the Study

The study adopts an observational methodology. Improvement of knowledge about CRPD and possible modifications of the attitude of those who participated in the intensive training on CRPD and human rights in mental health was measured with a before-after comparison.

Evaluation of the Razi hospital using the Quality Rights Tool Kit was compared with that obtained by the evaluation conducted in 2014 of the same Razi hospital using the same QualityRight Tool Kit [6].

### 2.3 Phases and Timing of the Action

The general timing of the project was established during a preliminary meeting in November 2017, between the team of the University of Cagliari and the team of the two Tunisian units, representing the Razi University Hospital of Manouba - Tunis and the CHU Mongi Slim La Marsa Hospital.

The World Health Organization, as an external partner to the project, was to provide two trainers (SVB from the WHO Collaborative Center in Lille, and MB, a WHO intern in Geneva) who would conduct the training together MGC and one expert for the discussion of the results (MFM from Columbia University).

The first operational phase of the study was the implementation of training in Tunis (from 12 to 17 February 2018).

The course had the general purpose of training participants on the principles of the CRPD and on the legal, social and health implications of applying the same convention. Also proposed on a practical level was the dissemination of knowledge on the use of tools (WHO-Quality Rights Tool Kit [5]) developed by WHO for the implementation of plans to improve the quality of rights in the practice of care services in mental health. This would allow the implementation of the subsequent phases of the program. As scheduled, 19 learners participated in the course: professionals (psychiatrists, psychologists, nurses, occupational therapists, speech therapists) from the two collaborative centers (Razi and La Marsa) as well as two jurists and two forensics from the University of Tunis, and two people who had experience in treating psychosocial disability.

The next phase of the study was the completion of the evaluation of an inpatient unit of Razi Hospital, which took place through an accreditation visit of the structure and interviews with staff, users and family members. The evaluation phase was prepared from 2 to 7 June 2018. The visit to the facilities was conducted by the project leader (MGC) with WHO staff (SVB and MFM) in collaboration with staff and family representatives. Data collection through the QualityRights Tool interview was conducted in the period from 1 July to 7 July 2018.

From 12 to 17 August, during a new work meeting, the results were codified, and two discussion sessions were held with staff, Tunisian assessment experts, WHO staff and family members. In the following months, the report was prepared and the discussion continued via Skype meetings and e-mails.

### 2.4 Tools

The tool used to verify the knowledge and attitudes of participants in the training in human rights and the CRPD was a questionnaire developed by the team of the World Health Organization that deals with the QualityRights study [5]. The questionnaire investigates the knowledge of the CRPD in general and the realization of the same in the field of mental health through 26 multiple-choice questions, The answers to the questionnaire were: I totally agree, I agree, I am neutral, I disagree and I totally disagree. The questionnaire was administered before the start of the training and at the end, so as to highlight the impact that the training had on learners’ knowledge and attitude.

As for the assessment of the unit of the Razi Hospital, the tool used was the QualityRights Toolkit [5]. The QualityRights Toolkit aims to support countries in assessing and improving the quality and respect for human rights in mental health and social care facilities. The QualityRights Toolkit contains 5 themes, taken from the CRPD, which are:

(1) The right to an adequate standard of living (Article 28 of the CRPD).

(2) The right to enjoy the highest achievable standard of physical and mental health (Article 25 of the CRPD).

(3) The right to exercise the legal capacity and the right to freedom and security of the person (Articles 12 and 14 of the CRPD).

(4) The right not to be subjected to torture or cruel, inhuman or degrading treatment or punishment, or to exploitation, violence, and abuse (Articles 15 and 16 of the CRPD).

(5) The right to live independently and be included in the community (Article 19 of the CRPD).

Each of the themes/rights in the toolkit is then divided into a series of standards, which in turn are divided into a series of criteria. The criteria are the basis for the quality assessment and respect for human rights. It is by means of criteria that the situation in the structures is assessed, through interviews, observations and reviews of the documentation. The evaluation of each criterion allowed those who carried out the evaluation to determine if a certain standard was reached. The standards, in turn, helped to determine if the general theme was implemented. The QualityRights Toolkit also provides detailed instructions on how to carry out the evaluation and how to report the results obtained; in fact, it provides the evaluation tools (the WHO QualityRights interview tool and the WHO QualityRights tool for document review and observation) and the tabs for the report. The interviews carried out in Razi hospital during 2018 took place in French with an operator who, if necessary, acted as a French / Arab interpreter. The interviews carried out in 2014 assessment took place in Arabic only.

### 2.5 Sample

The training sample was composed of 8 males and 11 females, whose average age was 41.4 ± 10.6 years. Participants included: 2 jurists/lawyers, 1 trainer, 2 managers, 8 health professionals, 4 university teachers, 2 attenders with both health professional and university teacher profiles. Each participant was free not to complete the questionnaire without having to present any justifications.

As regards the sample related to the evaluation of the Razi hospital, 20 random staff representatives (doctors, nurses, cleaning staff), 20 representatives of family members and 20 users were interviewed, for a total of 60 people. In the evaluation carried out in 2014, the sample was composed of 35 users, 18 representatives of family members and 35 representatives of the staff, for a total of 88 people.

### 2.6 Data Analysis

The analysis of the results on the questionnaire administered to participants in the training was performed with a one-way ANOVA statistical analysis. This analysis made it possible to calculate which questions showed a difference between the answers to the first administration (before training) with respect to the second (after training) and their statistical significance. The threshold value for the significance level was set at 0.05. The analysis was carried out with the Bonferroni correction since the high number of measures increased the probability of alpha errors.

As for the analysis of the results obtained from the evaluation of the Razi hospital with the QualityRights Toolkit, this was reported in the grid provided by the Toolkit.

The assessments relating to criteria, standards and topics were reported in the results grids with a rating scale divided into 4 levels:

- Completely achieved (A / F-Achieved Fully): it is evident that the criterion/standard/theme has been totally achieved.
- Partially achieved (A / P-Achieved Partially): it is clear that the criterion/standard/theme has been achieved, but improvements are needed.
- Problem that is starting to be addressed (A / I-Achievement initiated): it is clear that there has been a commitment to the realization of the criterion/standard/theme, but substantial improvements are needed.
- Not started (N / I-Not initiated): there is no evidence that something has been put in place for the realization of the criterion/standard/theme.

The results obtained in the 2014 evaluation were analyzed in the same way.

### 2.7 Ethical Aspects

The board of the Razi hospital in Tunisia approved this project. Informed consent was obtained from those who agreed to take part in the project.

## 3 RESULTS

For each of the 26 items of the questionnaire, Table **1** shows the average score and the standard deviation of answers reported at time T0 (pre-training) and T1 (post-training). The questionnaire was completed by 19 participants before the training, and by 15 participants at the end of the training.

Participants under training showed a general tendency to greater sensitivity towards the patient's point of view and against the use of coercive practices near the end of the training. Most items show a modification of the scores in this sense. However, a statistically significant before-after difference was achieved only in items: J – People who use mental health services should have the power to decide on their treatments (increased score P=0.004); M – People with psychosocial disabilities need someone to plan all their activities (decreased score P=0.049); N – The opinions of people with psychosocial disabilities should have more weight with regard to their treatments than the views of health professionals (increased score P=0.007); O – It is unacceptable to put pressure on users of a mental health service to take treatment they would not like (increased score P=0.04); T – Controlling users of mental health services is necessary to maintain order (decreased score P=0.014).

Tables **2**-**6** show the results, divided by theme, obtained following the evaluation of Razi hospital, carried out during 2018. Based on the results obtained in the individual criteria, it was possible to evaluate the score for the standards, and from the scores of the standards, it was possible to obtain the overall score for each theme, *i.e*., the score for specific rights of the CRPD related to each theme. With regards to Theme 1 (Table **2**) “The right to an adequate standard of living” (Article 28 of the United Nations Convention on the Rights of Persons with Disabilities, CRPD), only standard 1.4 (“Service users are given food, safe drinking water and clothing that meet their needs and preferences“) resulted as partially achieved, all other standards resulted initially achieved, except standard 1.7 (“Service users can enjoy fulfilling social and personal lives and remain engaged in community life and activities“) that was ‘not started’. With regards to Theme 2 (Table **3**) “The right to the enjoyment of the highest attainable standards of physical and mental health“ (Article 25 of the CRPD), standard 2.1 (“Facilities are available to everyone who requires treatment and support“) resulted fully achieved. The other standards were initially achieved; standard 2.3 (“Treatment, psychosocial rehabilitation and links to support networks and other services are elements of a service user-driven recovery plan and contribute to a service user’s ability to live independently in the community“) resulted in ‘not started’. Concerning the theme “The right to exercise legal capacity and the right to personal liberty and the security of persons“ (Articles 12 and 14 of the CRPD) (Table **4**), standard 3.4 (“Service users have the right to confidentiality and access to their personal health information“) was found partially achieved, while all the other standards were ‘not initiated’ except for standard 3.3 (“Service users can exercise their legal capacity and are given the support they may require to exercise their legal capacity“) which was partially achieved. As regards Theme 4, “Freedom from torture or cruel, inhuman or degrading treatment or punishment and from exploitation, violence and abuse“ (Articles 15 and 16 of the CRPD) (Table **5**), standard 4.4 (“No service user is subjected to medical or scientific experimentation without his or her informed consent“) was totally achieved, standard 4.3 (“Electroconvulsive therapy, psychosurgery and other medical procedures that may have permanent or irreversible effects, whether performed at the facility or referred to another facility, must not be abused and can be administered only with the free and informed consent of the service user“) was partially achieved; standards 4.1 (“Service users have the right to be free from verbal, mental, physical and sexual abuse and physical and emotional neglect“) and 4.5 (“Safeguards are in place to prevent torture or cruel, inhuman or degrading treatment and other forms of ill-treatment and abuse“) were partially achieved and standard 4.2 (“Alternative methods are used in place of seclusion and restraint as means of de-escalating potential crises“) was ‘not initiated’. All standards included in Theme 5 (“The right to live independently and be included in the community“ (Article 19 of the CRPD) (Table **6**) resulted ‘not initiated’.

Table **7** shows the synthetic score by theme and the comparison between the scores of 2014 and 2018 at Razi Hospital. In four themes, the scores were identical over time, but in Theme 2 (“The right to enjoyment of the highest attainable standards of physical and mental health“ - Article 25 of the CRPD), the score decreased from partially achieved to initiated.

## 4 DISCUSSION

Participants of the intensive one-week training on CRPD and human rights in mental health showed a significant improvement in knowledge or attitude, as reflected in a significant change in scores in 5 questions out of 26 of the questionnaire. These 5 questions have in common the participants’ 'beliefs about user control and users' freedom of choice. Although the difference reached statistical significance only in 19% of the questions, an improvement (in some cases at the limits of statistical significance) was also observed in many other questions, or in the clear majority, and it can be said that those who participated in the training are more willing to leave freedom of choice to those who use mental health services. It also appears to have changed to the belief that it is important not to try to control or replace the user since this can often prove harmful.

The data obtained from the 2018 assessment of the Razi hospital in Tunisia in accordance with QualityRights Toolkit show a condition in which the achievement of the rights declared by the CRPD is only partial or insufficient. Concerning the implementation of Article 28 of the CRPD, that is, the right to an adequate standard of living, the results clearly demonstrate that most of the standards show deficiencies and / or an initial level of achievement. The only standard that has been partially achieved relates to food, water, and clothing provided to users of the facility, while as regards, the standard on social life and participation of users in the community, it has been found that no efforts have yet been made to achieve it. Concerning the implementation of article 25 of the CRPD, that is, the right to enjoy the highest achievable level of physical and mental health, most of the standards are classified as objectives that are starting to be implemented. Only the standard regarding the availability of facilities for all those who require care and support is classified as fully achieved, while the standard of the presence of a plan for recovery and user participation in the drafting of the latter is classified as uninitiated. The achieving of rights provided for in Articles 12 and 14 of the CRPD, *i.e*., the right to exercise legal capacity and the right to personal freedom and personal security, show some delay. In this “theme”, the two standards, the first concerning preferences of users regarding their treatment and the second concerning procedures put in place to avoid detention and treatment without consent, are classified as unrealized. The third standard, regarding legal capacity, is classified as starting to be achieved. Only the last standard, which concerns the right to privacy and access to one's personal health information, is classified as partially achieved. The results concerning Articles 15 and 16 of the CRPD, *i.e*., the right to freedom from torture or from cruel, inhuman or degrading treatment or punishment and from exploitation, violence and abuse, are not univocal. In this theme, the first and last standards are classified as objectives that are being started. There is then a totally realized standard; No user is exploited for medical or scientific experimentation without his or her consent. The standard requiring alternative methods to insulation and restraint in the structure are unrealized. The standard concerning electroconvulsive therapy and its non-abuse is classified as partially realized. Article 19 of the CRPD, *i.e*., the right to live independently and be included in the community, is the one that shows the most negative results. In fact, all standards and all criteria have been classified as unrealized objectives.

Taken together, the evaluation shows significant delays in the implementation of the rights advocated by the CRPD. If in Themes 1, 3, 4 and 5, the evaluation shows no differences compared to 2014, it showed in Theme 2, an even lower level than four years before; from “achieved partially” in 2014 to “achievement initiated” in 2018 [6].

It must be kept in mind that the evaluation team in 2014 was composed in a different way. The evaluation was then carried out only by the structure’s staff, while in 2018, external experts were also involved and, above all, they were able to directly evaluate users and family members. This was not the case during the 2014 assessment and the different compositions may have increased the level of severity of the judgments.

However, the worsening specifically concerned items that the team members themselves complained of, especially in relation to the difficulty in obtaining drugs of primary necessity and to the decrease in staff that mainly involved people employed in rehabilitation and networking. Hence, the difficulty in the opening to the outside world and problems in the work of social inclusion.

It cannot be said that the scarcity of resources related to the serious economic and political crisis that Tunisia [7] is going through can be considered the only cause of the delays highlighted. However, it is likely that in a context of general crisis and uncertainty for the future, the scarcity of resources and the decrease in staff, in particular of professionals dedicated to psychosocial intervention [8, 9] may have been one of the factors that demotivated the team towards recovery and social inclusion [10-12].

An indirect demonstration of this demotivation emerges from the fact that the staff of Razi hospital has seen in recent years an impoverishment due to the departure of many professionals for jobs abroad.

An important fact is that the evaluation conducted in 2014 does not appear to have led to any improvement and it may be a demonstration that conducting an assessment without staff training and carrying out improvement plans may be ineffective.

## CONCLUSION

The evaluation conducted in 2018 reveals significant delays in the implementation of the rights advocated by the CRPD. In themes 1, 3, 4 and 5, the evaluation shows no differences compared to 2014, but as concerns Theme 2, we find an even lower level than four years before.

The scarcity of resources related to the serious economic crisis that Tunisia is going through cannot be considered the only cause of the delays highlighted. It is likely that in a context of general crisis and uncertainty for the future, the scarcity of resources and the decrease in staff (in particular of professionals dedicated to psychosocial intervention) may have been one of the factors demotivating the teamwork towards recovery and social inclusion.

However, the training on Quality Rights appears, at least in part, to have changed the knowledge and attitudes of many staff members, and this may open positive scenarios for the future. The commitment that staff and users have made in this action is another element that shows a desire for improvement.

The paper does not imply that we were expecting a change in the quality of care of the service, which is not the case because we are aware that assessments can only help to understand the level of the respect of human rights in a done facility and limited training, alone, does not allow to begin a process of change. The results and the needs emerged indicate that there is need of consolidated training throughout Tunisia (with the inclusion of all stakeholders) and specific transformation plans for the services would be required to see the change in human rights respect and quality of care – as was done in Gujarat and as is happening in other countries [13].

## Figures and Tables

**Table 1:** Results on QualityRights Questionnaire pre and post-training

**Item**	Mean±SD **t0**	Mean±SD **t1**	**F**	**P**
a- Knowledge and understanding of human rights can improve the quality of care in mental health services	4.94±0.22 (N=19)	4.93±0.24 (N=15)	0.016 df 1,32,33	0.900
B – Mental health workers can do a lot to improve the rights of people with mental disorders	4.70±0.45 (N=17)	4.86±0.33 (N=15)	1,328 df 1,32,33	0.258
C – People with severe mental disorders should consult their doctor before getting married	3.63±1.17 (N=19)	3.46±0.95 (N=15)	0.208 df 1,32,33	0.651
D - Much can be improved in mental health services without additional resources	4.00±0.66 (N=18)	4.07±0.96 (N=15)	0.063 df 1,31,32	0.803
E – People with dementia should live in structures where people could take care of them	3.15±1.53 (N=19)	2.33±1.19 (N=15)	2.911 df 1,32,33	0.098
F – People with psychosocial disabilities should not be employed in jobs that require contact with the public	2.00±1.07 (N=19)	1.80±0.90 (N=15)	0.336 df 1,32,33	0.566
G – Medicines are the most important factor in enabling people with mental disorders to get better	2.31±1.07 (N=19)	2.35±1.28 (N=14)	0.010 df 1,31,32	0.923
H – Taking your medicine is the most important factor in helping people with mental disorders get better	2.61±1.06 (N=18)	2.94±1.28 (N=13)	0.615 df 1,29,30	0.339
I – We only need to inspire hope once a person has recovered	2.55±1.06 (N=18)	1.93±0.85 (N=15)	3.072 df 1,31,32	0.090
J – People who use mental health services should have the power to decide on their treatments	3.26±1.01 (N=19)	4.20±0.65 (N=15)	9.763 df 1,32,33	0.004
K – Following the advice of other people who have experienced mental disorders is too risky	2.47±0.81 (N=19)	1.93±0.85 (N=15)	3.568 df 1,32,33	0.088
L – It is important to take tough positions with users of mental health services in order not to be manipulated	2.10±1.11 (N=19)	1.53±0.88 (N=15)	2.639 df 1,32,33	0.114
M – People with psychosocial disabilities need someone to plan all their activities	2.83±0.89 (N=18)	2.20±0.90 (N=15)	**4.170** df 1,31,32	0.049
N – The opinions of people with psychosocial disabilities should have more weight in regards to their treatments than the views of health professionals	2.55±0.68 (N=18)	3.46±1.14 (N=15)	**8.377** df 1,32,33	0.007
O – It is unacceptable to put pressure on users of a mental health service to take treatment they would not like	3.26±1.16 (N=19)	3.93±0.57 (N=15)	**4.185** df 1,31,32	0.049
P – People with mental disorders should not have important responsibilities	2.73±1.20 (N=19)	2.20±0.97 (N=15)	**1.927** df 1,31,32	0.175
Q – When people are unable to communicate, you have to make a decision based on what you think is best for them.	2.73±1.20 (N=19)	2.73±1.18 (N=15)	0.0001 df 1,31,32	0.9999
R – Health workers are in the best perspective to understand what they are capable of doing in life	2.57±1.09 (N=19)	2.20±0.97 (N=15)	**1.063** df 1,31,32	0.310
S – People with psychosocial disabilities have the right to make their decisions even if I don't agree with them	3.89±0.55 (N=19)	4.13±0.80 (N=15)	**1.073** df 1,31,32	0.308
T – Controlling users of mental health services is necessary to maintain order	3.26±1.06 (N=19)	2.33±1.01 (N=15)	**6.723** df 1,31,32	0.014
U – The use of isolation and restraint is necessary if users become threatening	3.10±1.16 (N=19)	2.46±1.20 (N=15)	**2.476** df 1,31,32	0.125
V – Isolation is not the ideal solution to manage a crisis	3.42±1.26 (N=19)	4.13±1.02 (N=15)	**3.134** df 1,31,32	0.086
W- The use of isolation and restraint negatively affects the therapeutic relationship between users of mental health services and staff	3.89±1.11 (N=19)	4.26±0.99 (N=15	**1.023** df 1,31,32	0.319
X - Locking up a person in a room is acceptable if the person presents a risk of injury or injury to others	3.36±0.92 (N=19)	2.86±1.30 (N=15)	**1.724** df 1,31,32	0.199
Y – Most people are not harmed if they are put on sedatives to defuse a tense situation	2.89±1.11 (N=19)	2.20±1.10 (N=15)	**3.265** df1,31, 32	0.080
Z – Involuntary hospitalization does more good than harm	2.61±1.20 (N=18)	2.46±1.08 (N=15)	**0.143** df 1,31,32	0.708

**Table 2:** Theme 1. - The right to an adequate standard of living (Article 28 of the United Nations Convention on the Rights of Persons with Disabilities, CRPD).

Standard	Score	Criterion					
1.1 The building is in good physical condition.		1.1.1	1.1.2	1.1.3	1.1.4		
	*A/I*	*A/I*	*A/I*	*N/I*	*A/I*		
1.2 The sleeping conditions of service users are comfortable and allow sufficient privacy.		1.2.1	1.2.2	1.2.3	1.2.4	1.2.5	1.2.6
	*A/I*	*N/I*	A/F	*A/I*	*A/I*	*A/I*	*N/I*
1.3 The facility meets hygiene and sanitary requirements.		1.3.1	1.3.2	1.3.3	1.3.4		
	*A/I*	*A/I*	A/P	A/F	*A/I*		
1.4 Service users are given food, safe drinking water and clothing that meet their needs and preferences.		1.4.1	1.4.2	1.4.3	1.4.4		
	A/P	A/P	A/P	*A/I*	A/P		
1.5 Service users can communicate freely, and their right to privacy is respected.		1.5.1	1.5.2	1.5.3	1.5.4	1.5.5	
	*A/I*	*N/I*	*N/I*	A/F	*A/I*		
1.6 The facility provides a welcoming, comfortable, stimulating environment conducive to active participation and interaction.		1.6.1	1.6.2	1.6.3	1.6.4		
	*A/I*	*A/I*	A/P	*N/I*	*N/I*		
1.7 Service users can enjoy fulfilling social and personal lives and remain engaged in community life and activities.	*N/I*	1.7.1 *N/I*	1.7.2 A/P	1.7.3 *N/I*	1.7.4 *N/I*	1.7.5 *N/I*	

**Table 3:** Theme 2. The right to enjoyment of the highest attainable standards of physical and mental health (Article 25 of the CRPD)

Standard	Score	Criterion					
2.1 Facilities are available to everyone who requires treatment and support.		***2.1.1***	***2.1.2***	***2.1.3***			
	A/F	A/P	A/F	A/F			
2.2 The facility has skilled staff and provides good-quality mental health services.		***2.2.1***	***2.2.2***	***2.2.3***	***2.2.4***	***2.2.5***	***2.4.6***
	*A/I*	N/I	N/I	A/P	A/P	*A/I*	N/I
2.3 Treatment, psychosocial rehabilitation and links to support networks and other services are elements of a service user-driven recovery plan and contribute to a service user’s ability to live independently in the community.		***2.3.1***	***2.3.2***	***2.3.3***	***2.3.4***	***2.3.5***	***2.3.6***
	N/I	A/I	N/I	N/I	N/I	A/I	A/I
2.4 Psychotropic medication is available, affordable and used appropriately.		***2.4.1***	***2.4.2***	***2.4.3***	***2.4.4***	***2.4.5***	
	A/I	A/I	N/I	A/P	A/I	N/I	
2.5Adequate services are available for general and reproductive health.		***2.5.1***	***2.5.2***	***2.5.3***	***2.5.4***	***2.5.5***	***2.5.6***
	A/I	A/P	A/P	A/P	N/I	A/I	A/P

**Table 4:** Theme 3. The right to exercise legal capacity and the right to personal liberty and the security of person (Articles 12 and 14 of the CRPD)

Standards	Score	Criterion						
3.1 Service users’ preferences regarding the place and form of treatment are always a priority.		***3.1.1***	***3.1.2***	***3.1.3***				
	N/I	A/I	N/I	N/I				
3.2 Procedures and safeguards are in place to prevent detention and treatment without free and informed consent.		***3.2.1***	***3.2.2***	***3.2.3***	***3.2.4***	***3.2.5***	***3.2.6***	
	N/I	N/I	N/I	N/I	A/F	N/I	N/I	
3.3 Service users can exercise their legal capacity and are given the support they may require to exercise their legal capacity		***3.3.1***	***3.3.2***	***3.3.3***	***3.3.4***	***3.3.5***	***3.3.6***	***3.3.7***
	A/I	A/I	A/I	N/I	A/I	A/I	N/	N/
3.4 Service users have the right to confidentiality and access to their personal health information.		***3.4.1***	***3.4.2***	***3.4.3***	***3.4.4***			
	A/P	A/F	N/I	A/F	N/I			

**Table 5:** Theme 4. Freedom from torture or cruel, inhuman or degrading treatment or punishment and from exploitation, violence and abuse (Articles 15 and 16 of the CRPD)

Standard	Score	Criterion					
4.1 Service users have the right to be free from verbal, mental, physical and sexual abuse and physical and emotional neglect.		***4.1.1***	***4.1.2***	***4.1.3***	***4.1.4***	***4.1.5***	
	A/I	A/P	A/I	A/I	N/I	N/I	
4.2 Alternative methods are used in place of seclusion and restraint as means of de-escalating potential crises.		***4.2.1***	***4.2.2***	***4.2.3***	***4.2.4***	***4.2.5***	
	N/I	N/I	N/I	N/I	N/I	A/P	
4.3 Electroconvulsive therapy, psychosurgery and other medical procedures that may have permanent or irreversible effects, whether performed at the facility or referred to another facility, must not be abused and can be administered only with the free and informed consent of the service user.		***4.3.1***	***4.3.2***	***4.3.3***	***4.3.4***	***4.3.5***	***4.3.6***
	A/P	A/F	A/F	A/F	A/F	N/I	A/F
4.4 No service user is subjected to medical or scientific experimentation without his or her informed consent.		***4.4.1***	***4.4.2***	***4.4.3***	***4.4.4***		
	A/F	A/F	A/F	A/F	A/F		
4.5 Safeguards are in place to prevent torture or cruel, inhuman or degrading treatment and other forms of ill-treatment and abuse.		***4.5.1***	***4.5.2***	***4.5.3***	***4.5.4***	***4.5.5***	***4.5.6***
	A/I	N/I	A/P	N/I	N/I	A/P	N/I

**Table 6:** Theme 5. The right to live independently and be included in the community (Article 19 of the CRPD)

5.1 Service users are supported in gaining access to a place to live and have the financial resources necessary to live in the community.		***5.1.1***	***5.1.2***	***5.1.3***
	N/I	N/I	N/I	N/I
5 .2 Service users can access education and employment opportunities.		***5.2.1***	***5.2.2***	***5.2.3***
	N/I	N/I	N/I	N/I
5.3 The right of service users to participate in political and public life and to exercise freedom of association is supported.		***5.3.1***	***5.3.2***	***5.3.3***
	N/I	N/I	N/I	N/I
5.4 Service users are supported in taking part in social, cultural, religious and leisure activities		***5.4.1***	***5.4.2***	***5.4.3***
	N/I	N/I	N/I	N/I

**Table 7:** Synthetic score by theme: comparison 2014-2018 at Razi Hspital

**Theme**	**Description**	**SCORE 2018**	**SCORE** **2014**
1	The right to an adequate standard of living (Article 28 of CRPD)	*A/I*	*A/I*
2	The right to the enjoyment of the highest attainable standards of physical and mental health (Article 25 of the CRPD)	A/I	A/P
3	The right to exercise legal capacity and the right to personal liberty and the security of person (Articles 12 and 14 of the CRPD)	*A/I*	A/I
4	The right to exercise legal capacity and the right to personal liberty and the security of person (Articles 12 and 14 of the CRPD)	*A/I*	*A/I*
5	The right to live independently and be included in the community (Article 19 of the CRPD)	*N/I*	*N/I*
