# Implementing WHO-Quality Rights Project in Tunisia: Results of an Intervention at Razi Hospital

**DOI:** 10.2174/1745017902117010008

**Published:** 2021-02-19

**Authors:** Mauro Giovanni Carta, Rym Ghacem, Myriam Milka, Olfa Moula, Nidhal Staali, Uta Uali, Ghassene Boukhari, Monica Mannu, Rym Refrafi, Souha Yaakoubi, Maria Francesca Moro, Marie Baudel, Simon Vasseur-Bacle, Natalie Drew, Michelle Funk

**Affiliations:** 1 Department of Medical Sciences and Public Health, University of Cagliari, Cagliari, Italy; 2 Razi Hospital, Tunis, Tunisia; 3 Mental Health Departement ,University Hospital Mongi Slim, Tunis, Tunisia; 4 Mailman School of Public Health, Columbia University, New York, NY, USA; 5 Department of Public Health, WHO, Geneva, Switzerland; 6 WHO Collaborating Centre for Mental Health Lille, Lille, France

## Correction

**Implementing WHO-Quality Rights Project in Tunisia: Results of an Intervention at Razi Hospital**

**Clinical Practice & Epidemiology in Mental Health, 2020, 16: 125-133 **

**Correction**

The corrections are provided and replaced online which is mentioned as under:

**Original**

The name of coauthor was Ghassene Bouakhari

**Corrected**

The name of coauthor has been revised as Ghassene Boukhari

